# Effects of high-intensity training on fatty infiltration in paraspinal muscles in elderly males with osteosarcopenia – the randomized controlled FrOST study

**DOI:** 10.1186/s12877-024-04736-5

**Published:** 2024-02-08

**Authors:** Kaja Kircher, Oliver Chaudry, Armin M. Nagel, Mansour Ghasemikaram, Michael Uder, Franz Jakob, Matthias Kohl, Wolfgang Kemmler, Klaus Engelke

**Affiliations:** 1 Institute of Medical Physics, Friedrich-Alexander-University of Erlangen-Nürnberg (FAU), Henkestr. 91, 91052 Erlangen, Germany; 2 Department of Medicine III, Friedrich-Alexander University of Erlangen-Nürnberg, University Hospital Erlangen, Ulmenweg 18, 91054 Erlangen, Germany; 3 Institute of Radiology, Friedrich-Alexander-University of Erlangen-Nürnberg and University Hospital Erlangen, Maximiliansplatz 3, 91054 Erlangen, Germany; 4 Faculty Medical and Life Sciences, University of Furtwangen, Neckarstrasse 1, 78054 Villingen-Schwenningen, Germany; 5 Bernhard-Heine-Center for Locomotion Research, University of Würzburg, Brettreichstrasse 11, 97074 Würzburg, Germany

**Keywords:** Osteosarcopenia, IMAT, HIRT, Dixon imaging, MRI, BMD

## Abstract

**Background:**

Osteosarcopenia is a common geriatric syndrome with an increasing prevalence with age, leading to secondary diseases and complex consequences such as falls and fractures, as well as higher mortality and frailty rates. There is a great need for prevention and treatment strategies.

**Methods:**

In this analysis, we used magnetic resonance imaging (MRI) data from the randomised controlled FrOST trial, which enrolled community-dwelling osteosarcopenic men aged > 72 years randomly allocated to 16 months of twice-weekly high-intensity resistance training (HIRT) or a non-training control group. MR Dixon imaging was used to quantify the effects of HIRT on muscle fat infiltration in the paraspinal muscles, determined as changes in muscle tissue, fat faction and intermuscular adipose tissue (IMAT) in the erector spinae and psoas major muscles. Intention-to-treat analysis with multiple imputation was used to analyse the data set.

**Results:**

After 16 months of intervention, 15 men from the HIRT and 16 men from the CG were included in the MRI analysis. In summary, no positive effects on the fat infiltration of the erector spinae and psoas major muscles were observed.

**Conclusions:**

The previously reported positive effects on lumbar spine bone mineral density (BMD) suggest that mechanotransduction induces tropic effects on bone, but that fat infiltration of the erector spinae and psoas major muscles are either irreversible or, for some unknown reason, resistant to exercise. Because of the beneficial effects on spinal BMD, HIRT is still recommended in osteosarcopenic older men, but further research is needed to confirm appropriate age-specific training exercises for the paraspinal muscles. The potential of different MRI sequences to quantify degenerative and metabolic changes in various muscle groups must be better characterized.

**Trial registrations:**

FrOST was approved by the University Ethics Committee of the Friedrich-Alexander University of Erlangen-Nürnberg (number 67_15b and 4464b) and the Federal Office for Radiation Projection (BfS, number Z 5–2,246,212 – 2017–002). Furthermore, it fully complies with the Declaration of Helsinki and is registered at ClinicalTrials.gov: NCT03453463 (05/03/2018). JAMA 310:2191–2194, 2013.

## Background

The age-related loss of skeletal muscle mass and function, known as sarcopenia, is a common problem in the elderly population. It is associated with physical disability, loss of independence, reduced quality of life, increased risk of morbidity and mortality, as well as frailty [1–4]. In combination with osteoporosis – referred to as osteosarcopenia – the negative effects of this syndrome are multiplied, resulting in a high rate of falls and fractures caused by the decline in muscle quality, muscle function and bone mineral density (BMD) [5, 6]. As previous studies have shown, maintaining independence, mobility and therefore muscle function seems to be linked to the amount of fat infiltration and the decrease in muscle tissue volume [7, 8].

IMAT – intermuscular adipose tissue – defined as the fat infiltrations within the fascial envelope of a muscle, is an indicator of muscle quality [9–11]. Several publications have shown a negative association between IMAT volume and physical function, muscle strength and power [7, 9, 12, 13]. Furthermore, fatty infiltration of muscle may be an early predictor of degeneration of muscle strength [14]. This suggests the importance of early and accurate detection of changes in muscle composition.

Up to 14.3% of the male community-dwelling population between 60–64 years have osteosarcopenia, with prevalence increasing with age [5]. In general, resistance training is effective in preventing or alleviating a wide range of age-related conditions in older people [2, 15, 16]. In particular, a combination of high-intensity resistance training (HIRT) and a high protein diet is an option for the prevention of sarcopenia and osteosarcopenia and their consequences [2, 13, 17]. This has been confirmed by several previous publications showing positive effects of HIRT on thigh muscle volume and strength [7, 9, 12, 18, 19]. Using the Franconian Osteopenia and Sarcopenia Trial (FrOST) in men with osteosarcopenia, we have also shown significant positive effects on thigh IMAT after 16 months of HIRT [2, 15, 20–22].

Unfortunately, exercise studies investigating effects on fat infiltration of paraspinal muscles are rare, despite their major impact on chronic back pain and their important role in spinal stability [23, 24]. Therefore, in this study we performed a further analysis using FrOST of the effect of HIRT on paraspinal muscle fat infiltration in community-dwelling men with osteosarcopenia. Based on previously published results for thigh muscles, we hypothesised a significant positive effect on IMAT volume and paraspinal muscle fat fraction in a HIRT versus a non-training control group after 16 months of exercise [19].

## Methods

Approved by the University Ethics Committee (number 67_15b and 4464b) and the Federal Office for Radiation Projection (BfS, number Z 5–2,246,212 – 2017–002) FrOST (Franconian Osteopenia and Sarcopenia Trial) was initiated by the Institute of Medical Physics of the Friedrich-Alexander University Erlangen-Nürnberg, Germany. All participants aged 72 years or older were diagnosed with sarcopenia and osteopenia and gave written informed consent. Participants were informed in advance about the detailed study procedure. FrOST fully complies with the Declaration of Helsinki and is registered at ClinicalTrials.gov: NCT03453463 (05/03/2018) [25].

### Participants

For recruitment, 180 men who had previously participated in the FranSO (Franconian Sarcopenic Obesity) project were selected and responded to the invitation [20]. All men were 72 years of age or older and belonged to the group of FranSO participants with the lowest quartile of skeletal muscle mass index (SMI). In FrOST, the following inclusion criteria were applied: (a) morphometric sarcopenia, represented by a skeletal muscle mass index (SMI) of ≤ 7.26 kg/m^2^, (b) osteopenia or osteoporosis of the lumbar spine or total hip according to WHO T-score criteria [26, 27]. Exclusion criteria were: (a) secondary osteoporosis, (b) pharmacologic therapy or disease affecting bone or muscle metabolism in the last 2 years, (c) hip fractures, (d) limitations or problems preventing vigorous exercise, (e) participation in resistance training in the last 2 years, (f) intake of > 60 g/d ethanol. Forty-three (43) of the 180 men met the FrOST inclusion and exclusion criteria and were willing to participate in the study. Participants were randomly assigned to a control group (CG, *n* = 22) or a training group (HIRT, *n* = 21) by a randomised draw prepared by a researcher not involved in this project. Neither the researcher nor participants knew the allocation in advance (“allocation concealment”). 5 HIRT participants and 2 CG participants were lost to follow-up. Two participants each in the HIRT and CG group withdrew from the study immediately after randomisation and were unable to undergo the MRI scan. A further three drop-outs in the HIRT had contraindications for MR-imaging.

Due to artefacts of the MR images, a further 4 participants in the CG and 1 participant in the HIRT group were lost and could not be included in the ITT analysis. Thus, 15 participants in the HIRT and 16 participants in the CG group were included in the statistical analysis. Further information about the recruitment process can be found in previous publications [21, 22].

### Interventions

HIRT participants completed two supervised resistance training sessions per week for a total period of 16 months. Participation was monitored by licensed instructors, the gym’s smart card system (Kieser-Training, Erlangen, Germany) and analysis of the training records. HIRT participants were provided with detailed training protocols. Details on the exact exercises and training concept have been reported previously [21]. Briefly, the resistance exercises focused on a high-intensity resistance training (HIRT) approach, using a single-set training with periods of high exercise intensity and explosive movement speed in the concentric phase. In total, 12–14 exercises per session were prescribed from a pool of 18 exercises (calf raises, leg press, leg extension, leg curls, adduction, abduction, hip extension, latissimus front pulleys, pull-overs, seated rowing, back extension, inverse fly, bench press, military press, lateral raises, butterfly with extended arms, crunches, lateral crunches). The absolute exercise intensity was adjusted by the number of repetitions (e.g. 5–7) and the associated work to failure [22]. The endpoints of the set were categorised as “complete momentary muscular failure”, “(self-determined) repetitions maximum” (RM), or “non-repetition maximum” (nRM). Regular repetition maximum tests (1 RM-tests) of leg and bench press were performed to monitor participant’s performance development.

After 12 weeks of familiarisation and conditioning, the exercise training was divided into 8–12 week phases that included two or three linearly periodised 4-week mesocycles, with each 4th week as a regeneration week. The relative intensity during the mesocycles varied between 60 and 85% 1RM. After 4 months of training, approximately 40–65% of the sets per session were performed to the maximum number of repetitions per set. Supersets and drop-sets were used to further intensify the exercise protocol, but no further changes to the training protocol were planned after 13–14 months of training.

Independent of the study procedures, all participants were asked to maintain their lifestyle, dietary intake and physical activity.

### Supplements

Participants of both groups (HIRT, CG) received cholecalciferol, calcium, and whey protein powder. Depending on their 25-OH vitamin D3 status, they received 10,000 IE per week (serum concentration less than 75 nmol/l) or 5000 IE per week (serum concentration between 76 and ≤ 100 nmol/l) (MYVITAMINS, Manchester, UK). Daily calcium supplementation was estimated based on questionnaires provided by Rheumaliga, Switzerland to meet with the recommended amount of 1000 mg/d calcium intake (Sankt Bernhard, Bad Dietzenbach, Germany).

The HIRT group consumed a total protein intake of 1.5–1.6 g/kg/day compared with 1.2–1.3 g/kg/day in the control group [28]. The protein powder (Active PRO80, inkospor, Roth, Germany) consists of whey protein with a chemical value of 156. One hundred grams contain 80 g of protein (10.4 g of Leucin) and 1200 mg of calcium. The amounts of protein supplements were determined on the basis of 4-day dietary protocols (Freiburger Nutrition Record, Nutri-Sience, Hausach, Germany). The protocols were then evaluated, and participants were given supplements to individually achieve the cumulative total protein intake indicated above, following the current recommendations of the PROT-AGE study group.

### Assessments

All assessments were standardised to ensure high reproducibility and to avoid bias [2, 15, 20–22]. Data acquisition was performed by the same research assistants or imaging technologists at the same time of the day (± 2 h) and in the same locations. The research assistants performing the MRI scan and the medical imaging expert analysing the scan were blinded to the status of the participants (HIRT or CG). No strenuous physical activity or exercise was allowed 48 h before the assessments.

For logistic reasons, baseline MR scans were performed 5–6 weeks after the start of the intervention i.e., immediately after the 4 week familiarisation period. Follow-up MR scans were performed 4 weeks before the intended end of the intervention, during the last regeneration week of the penultimate mesocycles.

Body height was measured with a Holtain stadiometer (Crymych Dyfed, Great Britain), body mass with the scale function of a direct-segmental multifrequency bioimpedance analysis (DSM-BIA; InBody 770, Seoul, Korea) and body composition with dual energy X-ray absorptiometry (DXA, QDR 4500a, Discovery-upgrade, Hologic Inc., Bedford, USA). At baseline, participants completed a standardised questionnaire covering (a) demographic parameters, (b) diseases, (c) medication, (d) operations, (e) physical limitations, (f) falls, injuries and low trauma fractures and (g) lifestyle, including physical activity and exercise. A comparable questionnaire was completed at the end of the study, mainly to detect changes in variables that might affect the study results. Participants were asked to list their medications and medical conditions to ensure accuracy of the responses. The completed questionnaires were carefully checked for consistency, completeness, and accuracy in close cooperation between the primary investigator (WK) and the participants.

### MR Imaging

MR imaging of the lumbar spine was performed using a 3 T scanner (MAGNETOM Skyra-fit, Siemens Healthineers AG, Erlangen, Germany) in combination with a body surface coil. To define the region of interest (ROI), a coronal fast spoiled gradient echo scout scan was performed to locate the scan area, which completely covered the L2 to L4 vertebrae. An axial T1-weighted turbo spin echo and a volumetric interpolated breath-hold 6-point Dixon sequence were acquired. T1 images were acquired with the following parameters: voxel size 0.4 × 0.4 x 3mm^3^, slice gap 0 mm, matrix size 512 × 272, 44 slices per scan. The Dixon technique provides proton density fat fraction (FF) maps [19, 29]. In these maps, the intensity value of a given voxel encodes the FF in steps of 0.1%, i.e. an intensity value of 0 (1000) is equivalent to a FF of 0.0% (100.0%) [24, 30]. Dixon images were obtained with a voxel size of 0.8 × 0.8 x 3mm^3^, a slice gap of 0 mm and a matrix of 320 × 160, resulting in 36 slices per scan. Dixon images covered a length of 108 mm and T1w images of 132 mm.

### MR Image analysis

**Fig. 1:**
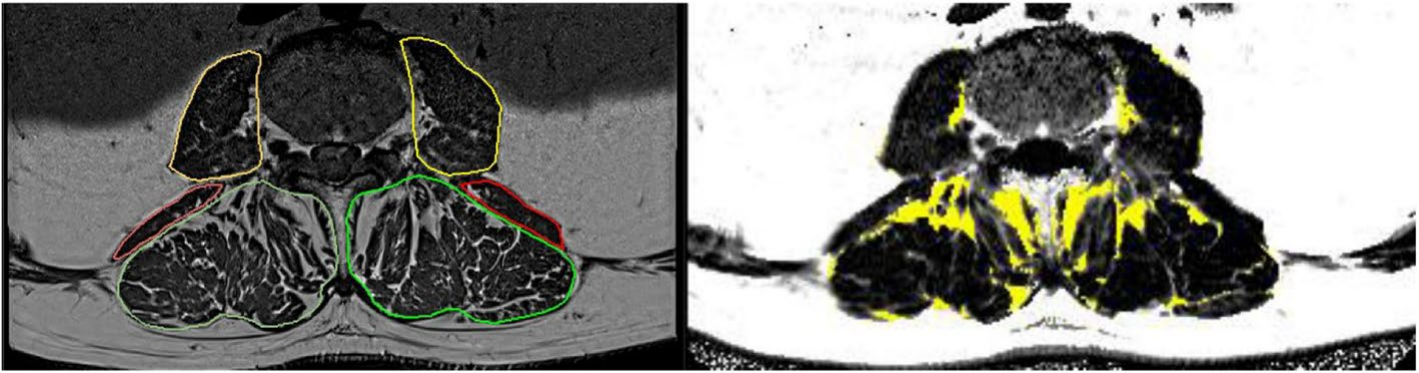
Left: Segmentation of T1-weighted MR images. The lines represent the fascial envelope of M. psoas (yellow), M. erector spinae (green) and M. quadratus lumborum (red). Right: IMAT within the fascial envelope in a Dixon MRI

The T1 scans were used to identify the axial slice between L3 to L4 that best represented the paravertebral muscles. In this slice, the fasciae of left and right psoas major and erector spinae were manually contoured using ImageJ (version 1.51) (Fig. 1). The area enclosed by the fascia multiplied by the slice thickness gives the intra-fascial volume (IF). In a second step, the resulting segmentation masks were registered to the Dixon FF images. Each muscle was then divided into muscle tissue (MT) and intermuscular adipose tissue (IMAT) using a subject-specific threshold [31]. This threshold was determined from a logarithmically scaled histogram of the FF values of the VOI, and the minimum of this histogram was used as the threshold to separate MT from IMAT in the selected slices as described previously [32, 33]. Finally, the volume and FF of IF, volume of the IMAT and volume and FF of MT were determined. Analysis was performed using MIAF (Medical Image Analysis Framework, University of Erlangen).

### Statistical analysis

We used intention-to-treat (ITT) analysis, which included all participants randomly assigned to the study arms (HIRT vs. CG), regardless of loss to follow-up, compliance, or confounding aspects, except of 6 individuals in the HIRT and 6 individuals in the CG who were excluded with completely missing or invalid MRI scans (baseline and follow-up). We applied multiple imputation (ITT) using R statistics software (R Development Core Team Vienna, Austria) in combination with Amelia II [34]. We used the full dataset for multiple imputations and repeated imputation 100 times. As confirmed by the over-imputation diagnostic plots (“observed versus imputed values”) provided by Amelia II, imputation worked well in all cases. We checked the normal distribution of the data using statistical and graphical tests (qq-plots). All within-group changes in the outcomes of interest were analysed using dependent t-tests. To compare the corresponding changes between CG and the HIRT (i.e. time-group interactions = effects), we applied an ANCOVA adjusting for baseline data of the outcome using group as a covariate. Within- and between-imputation variances were analysed using the approach suggested by Rubin and Barnard and Rubin [35, 36]. We consistently used 2-tailed tests and accepted significance at *p* < 0.05.

## Results

**Fig. 2:**
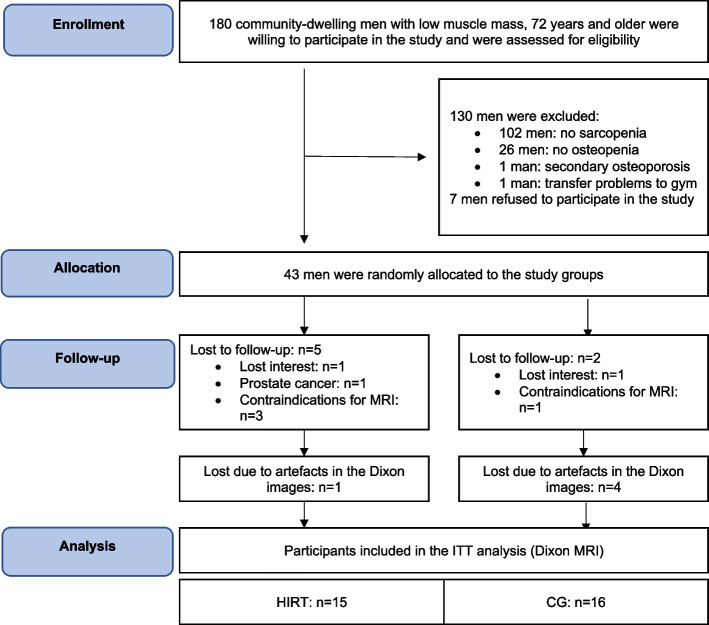
Selection process of patients included in the final analysis

Baseline characteristics are shown in Table 1. Apart from protein intake prior to supplementation, there were no significant differences between the two study groups at baseline [2]. As shown in Fig. 2, 15 HIRT men (71%) and 16 CG men (73%) with MR data were included in the statistical analysis [19]. The attendance rate of the HIRT group was very high (95 ± 5%). In parallel, compliance with whey-protein, calcium and Vit-D supplementation, which was monitored by telephone calls, delivery logs and personal interviews, showed an overlap of 82% (calcium) to 94% (protein) between the prescribed doses and the amount of supplements taken by the participants. No adverse effects or injuries were observed during the training sessions. Biweekly telephone calls revealed no changes in physical activity or exercise in the CG. At the same time, no changes in parameters (e.g. diseases, medication, and lifestyle) that could have confounded our results were identified through the follow-up questionnaires or personal interviews.

**Table 1:** Baseline characteristics of exercise and control groups

Variable	HIRT (*n* = 21)	CG (*n* = 22)	*p*
Age [years]	77.8 ± 3.6	79.2 ± 4.7	.262
BMD Lumbar Spine [mg/cm^2^]	1.054 ± .142	0.987 ± .115	.140
BMD Femoral Neck [mg/cm^2^]	0.894 ± .084	0.869 ± .094	.364
Body Mass Index [kg/m^2^]	25.0 ± 3.0	24.5 ± 1.9	.515
Calcium Intake (mg/d)^f^	802 ± 226	833 ± 282	.636
Energy Intake [MJ/d]^e^	8.84 ± 1.71	9.39 ± 2.42	.407
Exercise per week [min]	59 ± 56	46 ± 52	.780
Fat intake [g/d]	88.4 ± 33.5	81.1 ± 21.6	.208
LLFDI [Index] ^c^	1.51 ± 0.74	1.44 ± 0.53	.727
Physical activity [Index]^a^	4.45 ± 1.32	4.15 ± 1.53	.490
Protein Intake [g/kg/d]^e^	1.10 ± 0.25	1.29 ± 0.34	**.043**
Three (3) or more diseases [n]^b^	10	12	.826
Total Body Fat [%]	28.6 ± 5.8	30.5 ± 6.8	.330
25 (OH)D [nmol/l]^d^	43.8 ± 17.5	54.0 ± 21.1	.126

^a^scale from (1) “very low” to (7) “very high” (27)

^b^using the ICD-10 based disease cluster of Schäfer et al. [37]

^c^Late Life Function Disability Instrument [38]: scale from (1) “no problem” to (5) “impossible”

^d^Roche Diagnostics, Mannheim, Germany

^e^as determined by a 4-day dietary record

^f^ as determined by a Calcium Questionnaire provided by Rheumaliga, Switzerland

Tables 2 and 3 show the results for the psoas major and Tables 4 and 5 for the erector spinae. All tables show the absolute changes at 16 months for each group separately, as well as the differences between HIRT and CG groups at baseline and the absolute changes.

**Table 2:** Right psoas muscle—baseline and absolute changes after 16 months

**Psoas right**		**Baseline MV (SD)**	**Absolut change MV (SD)**	p*
**IF volume [cm**^**3**^**]**	CG	3.05 (3.69)	-0.013 (0.14)	.678
	HIRT	3.17 (2.65)	-0.047 (0.17)	.216
	p**	.637	.560	
**IF FF [%]**	CG	12.5 (19.35)	0.272 (3.63)	.739
	HIRT	11.3 (21.6)	0.981 (3.97)	.279
	p**	.482	.674	
**IMAT volume [cm**^**3**^**]**	CG	0.124 (0.52)	0.014 (0.07)	.448
	HIRT	0.130 (0.52)	-0.006 (0.08)	.744
	p**	.881	.45	
**MT volume [cm**^**3**^**]**	CG	2.71 (2.93)	-0.007 (0.14)	.819
	HIRT	2.88 (2.52)	-0.053 (0.18)	.188
	p**	.446	.456	
**MT FF [%]**	CG	6.85 (8.28)	0.246 (2.16)	.613
	HIRT	6.05 (10.53)	0.677 (2.34)	.209
	p**	.333	.890	

p** Between-group differences (*CG* vs *HIRT*), p* Within-group differences (*FU* vs *BL*), *IF* intra-fascial, *FF* fat fraction, *IMAT* intra-muscular adipose tissue, *MT muscle tissue*, *CG* control group, *HIRT* exercise group, *Baseline* first baseline measurements without any interventions, *Δ Absolut* absolute change from baseline to follow-up measurements after 16 months, *MV* mean value, *SD* mean standard deviation, significant *p* values are shown in bold

**Table 3:** Left psoas muscle—baseline and absolute changes after 16 months

**Psoas left**		**Baseline MV (SD)**	**Absolut change MV (SD)**	*p
**IF volume [cm**^**3**^**]**	CG	3.15 (3.555)	-0.026 (0.167)	.489
	HIRT	3.18 (2.655)	-0.017 (0.198)	.695
	p**	.905	.912	
**IF FF [%]**	CG	13.46 (26.1)	0.762 (3.62)	.353
	HIRT	13.72 (36.1)	0.488 (3.798)	.569
	p**	.921	.820	
**IMAT volume [cm**^**3**^**]**	CG	0.143 (0.765)	0.027 (0.077)	.147
	HIRT	0.183 (0.93)	-0.017 (0.086)	.394
	p**	.567	.066	
**MT volume [cm**^**3**^**]**	CG	2.78 (3.0)	-0.044 (0.144)	.180
	HIRT	2.80 (2.88)	0.01 (0.167)	.776
	p**	.927	.275	
**MT FF [%]**	CG	7.38 (8.19)	0.343 (2.79)	.585
	HIRT	6.46 (11.25)	0.997 (3.03)	.153
	p**	.285	.809	

p** Between-group differences (*CG* vs *HIRT*), p* Within-group differences (*FU* vs *BL*), *IF* intra-fascial, *FF* fat fraction, *IMAT* intra-muscular adipose tissue, *MT muscle tissue*, *CG* control group, *HIRT* exercise group, *Baseline* first baseline measurements without any interventions, *Δ Absolut* absolute change from baseline to follow-up measurements after 16 months, *MV* mean value, *SD* mean standard deviation, significant *p* values are shown in bold

**Table 4:** Right erector spinae muscle—baseline and absolute changes after 16 months

**Erector spinae right**		**Baseline MV (SD)**	**Absolut change MV (SD)**	*p
**IF volume [cm**^**3**^**]**	CG	7.75 (3.78)	-0.155 (0.347)	.059
	HIRT	7.89 (5.985)	-0.051 (0.342)	.508
	p**	.730	.305	
**IF FF [%]**	CG	24.58 (39.65)	0.568 (2.759)	.364
	HIRT	20.40 (27.59)	1.45 (2.77)	.**027**
	p**	.137	0.063	
**IMAT volume [cm**^**3**^**]**	CG	0.95 (2.61)	0.056 (0.248)	.321
	HIRT	0.79 (1.94)	0.051 (0.243)	.351
	p**	.392	.903	
**MT volume [cm**^**3**^**]**	CG	5.82 (5.45)	-0.137 (0.31)	.059
	HIRT	6.23 (4.14)	-0.147 (0.315)	**.049**
	p**	.296	.677	
**MT FF [%]**	CG	9.72 (11.25)	-0.109 (1.166)	.676
	HIRT	8.14 (10.13)	0.675 (1.20)	.**019**
	p**	.077	**0.015**	

p** Between-group differences (*CG* vs *HIRT*), p* Within-group differences (*FU* vs *BL*), *IF* intra-fascial, *FF* fat fraction, *IMAT* intra-muscular adipose tissue, *MT muscle tissue*, *CG* control group, *HIRT* exercise group, *Baseline* first baseline measurements without any interventions, *Δ Absolut* absolute change from baseline to follow-up measurements after 16 months, *MV* mean value, *SD* mean standard deviation; significant *p* values are shown in bold

**Table 5:** Left erector spinae muscle—baseline and absolute changes after 16 months

**Erector spinae left**		**Baseline MV (SD)**	**Absolut change MV (SD)**	*p
**IF volume [cm**^**3**^**]**	CG	7.84 (4.545)	-0.116 (0.41)	.216
	HIRT	8.00 (6.255)	-0.098 (0.414)	.297
	p**	.725	.785	
**IF FF [%]**	CG	25.71 (39.96)	1.00 (3.24)	.176
	HIRT	22.54 (39.56)	1.27 (3.375)	.105
	p**	.329	.327	
**IMAT volume [cm**^**3**^**]**	CG	1.03 (2.88)	0.080 (0.252)	.167
	HIRT	0.93 (3.195)	0.059 (0.266)	.325
	p**	.695	.819	
**MT volume [cm**^**3**^**]**	CG	5.74 (6.075)	-0.111 (0.468)	.297
	HIRT	6.07 (4.635)	-0.191 (0.482)	0.09
	p**	.453	.485	
**MT FF [%]**	CG	10.00 (10,22)	0.204 (2.133)	.671
	HIRT	8.97 (11.84)	0.659 (2.214)	.194
	p**	.255	.239	

p** Between-group differences (*CG* vs *HIRT*), p* Within-group differences (*FU* vs *BL*), *IF* intra-fascial, *FF* fat fraction, *IMAT* intra-muscular adipose tissue, *MT muscle tissue*, *CG* control group, *HIRT* exercise group, *Baseline* first baseline measurements without any interventions, *Δ Absolut* absolute change from baseline to follow-up measurements after 16 monthsm, *MV* mean value, *SD* mean standard deviation; significant *p* values are shown in bold

At baseline, no significant differences were observed between CG and HIRT for any of the parameters listed in Tables 2, 3, 4 5. After 16 months of intervention, we observed significant effects only for the right erector spinae (Table 4). In the HIRT group, IF (*p* < 0.03) and MT FF (*p* < 0.02) increased, and MT volume (*p* < 0.05) decreased. The absolute change in MT FF at 16 months was higher in the HIRT group than in the CG. In contrast, no significant changes were observed in the left erector spinae (Table 5) or in the psoas major (Tables 2 and 3).

Although not significant, it is interesting to note that muscle tissue FF increased in all four muscles in the HIRT and the CG, with the exception of the right erector spinae of the CG, and that the increases were greater in the HIRT than in the CG. Similarly, muscle tissue volume decreased, and IMAT volume increased in both groups, particularly in the erector spinae.

We did not perform a Bonferroni adjustment in this study. Only four out of 80 p-values indicated significance but this exactly the number to be expected at a 0.05 level (0.05*80 = 4). Consequently, they were interpretated as significant by chance.

## Discussion

In this paper we investigated the effect of HIRT on fat infiltration of paraspinal muscles. The analysis was equivalent to previously reported analysis of the effect on fat infiltration in the thigh muscle [19]. Surprisingly, and in contrast to the results observed in the thigh, the 16 month of high intensity resistance training had no effect on IMAT in paraspinal muscles. Thus, the hypothesis outlined in the introduction must be completely rejected.

It seems unlikely that the negative training results observed in the paraspinal muscles can be attributed to the small sample size, because in the same cohort there were significant positive training effects of 15% on thigh intermuscular adipose tissue (IMAT) volume, which increased significantly in the CG and remained stable in the HIRT group. In parallel, the IF fat fraction differed significantly between the groups (changes, EG: 0.77% vs. CG: 7.7%). In the thigh, MT-FF increased significantly in both groups, but the increases were smaller in HIRT than in CG. The differences between the groups were not significant, whereas in the spine increases in the CG were only about 30% of those in HIRT. Furthermore, in the same cohort, there was a significant positive training effect on lumbar spine bone mineral density [21]. In addition, the same MR scan protocol and an almost identical image analysis procedure (single slice in the spine versus multiple slice analysis in the thigh) was used.The HIRT used in FrOST was consisted of a single-set resistance exercise protocol performed on machines, focusing on high effort and intensity. Such a program has been recommended for older people [2, 16, 18, 39, 40]. However, to our knowledge, no previous studies have compared the effects of HIRT on thigh and paraspinal muscles in the same cohort. Interestingly, the effects of training on spinal muscles in older people have also been limited in other studies. In a cohort of men and women with low back pain (mean age 53 years), no significant training effects were found on muscle size or fatty infiltration of the erector spinae or multifidus using T1-weighted images centered on L4. Their training consisted of 10 weeks of high-intensity resistance training on machines in combination with a diet [11]. The study did not use Dixon sequences and therefore did not assess muscle tissue and IF FF. Results for fat infiltration measures were similar to IMAT volume measures in the FrOST study.

In contrast, in a younger cohort of men and women with LBP and a mean age of 40 years, significant reductions in fat infiltration of the erector spinae and multifidus were measured on T2-weighted images centered between L3/L4 as well as between L4/L5 after 16 weeks of free weight based resistance training [41]. Thus, training effects on muscle fat infiltration may largely depend on the age of the exercising subjects.

The chronification of inflammatory processes and fatty infiltration with age is likely to result in the generation of true adipocytes whose differentiation cannot be reversed by exercise. It is known from other clinical situations and from preclinical models that the reversibility of fatty infiltration depends on the time between lesion and reconstruction. For example, in a mouse model of delayed rotator cuff repair, significant muscle atrophy and fatty infiltration were observed when the time between lesion and surgery was too long [42]. Insight into the metabolism of fatty infiltration may also be gained from non-alcoholic fatty liver disease (NASH), which causes liver steatosis and, in its late stage mostly irreversible fibrosis and cirrhosis. On the other hand, in the early stages in children and adolescents, NASH can be reduced by exercise [43–45].

Exercises performed in FrOST also included muscle groups of the thigh and the spine, but it must be considered that exercises performed in FrOST may not match the complexity of functions and movements of the paraspinal muscles [19]. The erector spinae are involved in spinal rotation and extension as part of the lumbar erector spinae flexion-relaxation phenomenon [46]. Therefore, despite the wide variety of spinal exercises, not all parts of this muscle may have been effectively trained. There were also no positive effects of exercise training on the psoas major, but the reasons for this may be different, as age-related changes in IMAT and muscle tissue in the psoas are smaller in men than in the erector spinae [47].

In the FrOST cohort, the relative IMAT volume (IMAT volume / IF volume) was 0.12 for the erector spinae at baseline, about twice as high as in the thigh with a ratio of 0.07 [19]. This translated into an average IF FF of 23.3% in the erector spinae versus 16.9% in the thigh. Whether these differences are caused by sarcopenia needs to be further investigated, but in healthy men. The age-related decrease of IMAT volume / IF volume and the increases in IF FF are also much higher in the erector spinae compared to the thigh [47]. Similar age-related decreases in IF FF for the erector spinae were reported by Hoppe et al. (2021) in a mixed cohort of men and women. It should also be considered that differences in muscle fiber composition between the two muscle groups, thigh and paraspinal, may also be important [48]. Gauber et al. (2016) reported a higher proportion of type I fiber in paraspinal muscles compared to other skeletal muscles [24]. As described in the review by Kara et al. (2021), type II muscle fibers are the first to be affected by sarcopenia, so paraspinal muscles may be one of the last muscle groups to be affected by sarcopenia [49].

The spinal results observed in our analysis actually reflect age-related changes better than the effects of exercise training. It is interesting to note that some studies confirmed that high IMAT at baseline reduces the muscle response to exercise, which could be another explanation for the paraspinal results observed in FrOST [7, 12, 18]. This important point needs to be addressed in future studies, as in FrOST muscle strength assessments of the spine have not been performed, which is a limitation of the current study. Nevertheless, as discussed above, a mechanotransduction effect on bone was observed. Perhaps the potential of MRI Dixon sequences to monitor exercise effects on muscle may be limited in case of a chronic and prolonged inflammatory process. Other sequences such as T2 maps to quantify the amount of inflammation may be more sensitive in this case. Another limitation of the study was the MRI analysis, which was only performed in a single slice. An approach such as the STAR thigh adjustment ratio could be a solution to this limitation by using BMI to put the information obtained by local MRI into a whole-body context. However, cut-off values would need to be defined in a future study [49].

## Conclusion

In conclusion, HIRT had a positive effect on IMAT in the thigh, but not in the erector spinae or psoas major. However, we recommend the use of our HIRT program in osteosarcopenic community-dwelling elderly, as it prevented further increase in IMAT and FF in thigh muscles, whose degeneration is a high-risk factor for falls and therefore for fractures. In addition, previous publications from the FROST trial showed significant positive effects on the sarcopenia Z-score, SMI, and handgrip strength [2, 19]. Even without IMAT reducing effects on the paraspinal muscles, HIRT showed secondary beneficial effects by increasing BMD, which supports fractureprevention. Thus, some mechanotransduction should have occurred even through structurally altered muscle tissue. Further research is needed to re-evaluate appropriate age-specific training exercises for erector spinae and other paraspinal muscles, as well as the inverse phenomena seen in the two different muscle groups. It is also important to better characterise the potential of different MRI sequences to quantify degenerative and metabolic changes in different tissues, particularly in view of future studies on the effects of senolytic strategies, which, if successful, will need to address tissue structural changes [50–52].

## Data Availability

The datasets used and/or analysed during the current study are available from the corresponding author on reasonable request.

## Abbreviations

BMD: Bone mineral density
CG: Control group
EG: Exercise group
FF: Fat fraction
FrOST: Franconian Osteopenia and Sarcopenia Trial
HIRT: High intensity resistance training
IF: Intra-fascial volume
IMAT: Intermuscular adipose tissue
ITT: Intention-to-treat
LBP: Lower back pain
MRI: Magnetic resonance imaging
MT: Muscle tissue
NASH: Non-alcoholic fatty liver disease
Nrm: Non-repetition maximum
RM: Repetitions maximum
SMI: Skeletal muscle mass index
LLFDI: Late life function and disability instrument
